# Validation of suitable reference genes by various algorithms for gene expression analysis in *Isodon rubescens* under different abiotic stresses

**DOI:** 10.1038/s41598-022-22397-5

**Published:** 2022-11-15

**Authors:** Conglong Lian, Bao Zhang, Jingfan Yang, JinXu Lan, Hao Yang, Kaihua Guo, Jingjing Li, Suiqing Chen

**Affiliations:** School of Pharmacy, Henan University of Traditional Chinese Medicine, Boxue Road, Jinshui District, Zhengzhou, 450046 China

**Keywords:** Gene expression analysis, Plant molecular biology

## Abstract

*Isodon rubescens* (Hemsley) H. Hara (Lamiaceae) is a traditional Chinese medicine plant that has been used to treat various human diseases. Oridonin is one of the main active ingredients, and the route of its molecular biosynthesis remains to be determined. The study of gene expression patterns can provide clues toward the understanding of its biological functions. The selection of suitable reference genes for normalizing target gene expression is the first steps in any quantitative real-time PCR (RT-qPCR) gene expression study. Therefore, validation of suitable reference genes is necessary for obtaining reliable results in RT-qPCR analyses of *I. rubescens*. Here, 12 candidate reference genes were chosen, and their expression stability in different tissues of *I. rubescens* and in leaves under different abiotic stresses (NaCl, dehydration, SA, MeJA, and ABA) was evaluated using the ∆Ct, NormFinder, GeNorm, BestKeeper, and RankAggreg statistical tools. Analysis using the comprehensive tools of RankAggreg algorithm showed that *GADPH*, *18S* and *eIF* were stably expressed in different tissues; *UBQ, Apt,* and *HIS*; *Cycl, UBQ,* and *PP2A*; *GADPH, 18S,* and *eIF*; *eIF, UBQ,* and *PP2A; TUB, Cycl,* and *UBQ*; were the best three candidate reference genes for the samples of Dehydration, NaCl, SA, MeJA, and ABA treatment, respectively. While for the concatenated sets of ND (NaCl and dehydration) and SMA (SA, MeJA, and ABA), *UBQ*, *HIS*, and *TUA; UBQ*, *eIF* and *Apt* were the three appropriate candidate reference genes, respectively. In addition, the expression patterns of *HMGR* in different tissues and under different treatments were used to confirm the reliability of the selected reference genes, indicating that the use of an inappropriate reference gene as the internal control will cause results with a large deviation. This work is the first study on the expression stability of reference genes in *I. rubescens* and will be particularly useful for gene functional research in this species.

## Introduction

*Isodon rubescens* (Hemsley) H. Hara is a perennial subshrub belonging to a genus of the Lamiaceae family. The dry aerial portions of the *I. rubescens* plant are named Rabdosiae rubescentis herba, which have been used as a traditional Chinese medicine plant to treat various human diseases^1^. Rabdosiae rubescentis herba tastes bitter and slightly sweet, with clearing heat and detoxification, promoting blood circulation and relieving pain, and it is used for sore throats, scar tissue, and snake bites^1^. Modern pharmacological studies have shown that the main active ingredients, especially the oridonin of *I. rubescens*, provide it with a significant curative effect for breast cancer^2^, esophageal cancer^3^, laryngeal cancer^4^, colon cancer^5^, and also as an anti‐tumor agent^6^. Therefore, as an anti-tumor plant with good pharmacological activity and low hepatorenal toxicity, *I. rubescens* has been of significant interest to researchers studying its pharmacological efficacy. With the development and advancement of molecular pharmacognosy, more and more attention has been paid to the functional study of key genes in the synthesis and regulation of the main active ingredient in *I. rubescens*.

Until now, due to the importance of the main active ingredient of *I. rubescens*, several studies have investigated the expression of genes that are potentially involved in the biosynthesis of oridonin by using real-time quantitative reverse-transcriptase polymerase chain reaction (RT-qPCR) techniques^7^. In particular, with the emphasis on gene research of Chinese herbal medicines, the genes function of genes related to specific medicines are becoming more scrutinized. The screening of internal reference genes of Chinese herbal medicines has also made some progress, such as with *Panax ginseng*^8^, *Salvia miltiorrhiza*^9^, and *Ganoderma lucidum*^10^. Therefore, with the increase of studies on *I. rubescens* genes, it is of great significance to study the screening of its internal reference genes.

In the study of gene expression, RT-qPCR has been widely used as a powerful technique to determine the gene expression levels in different samples, due to its high sensitivity, efficiently, specificity, and reproducibility^11^. However, the accuracy of RT-qPCR results strongly depends on the precise normalization of the transcripts using stably expressed reference genes^12^. Reference genes are considered to be those with constant expression in all samples of the organism, including different tissues, or under different treatments, or at different developmental stages. Previous studies have shown that the common reference genes in many plants are those coding for elongation factor 1-α (*EF-1α*)^13^, actin (*ACT*)^14^, glyceraldehyde-3-phosphate dehydrogenase (*GADPH*)^15^, and 18S ribosomal RNA (*18S*)^16^, to name a few. However, it can be seen from previous studies that there is no universal reference gene across different species, and the most stable reference genes are different in different plants or under different treatments of the same plant^8^. And the use of inappropriate reference genes can result in target gene expression profile bias. Therefore, to get accurate and reliable gene expression profiles, it is important to identify the optimum reference genes among different tissues and treatments when considering evaluation by RT-qPCR.

In order to better screen out the most suitable internal reference genes, various statistical algorithms are used to calculate or evaluate the stability of reference genes in a set of samples. Based on previous reports, the main calculation tools for qRT-PCR data normalization included the ∆Ct method^17^, geNorm^18^, NormFinder^19^, and BestKeeper^20^. These methods select the most suitable gene or gene combination based on the calculation of expression stability for normalization. In addition, the calculation tools listed above use different algorithms and therefore might generate different stability ranking results^21,22^. RankAggreg, an R package for weighted rank aggregation, was used in a comprehensive sequencing study on genetic stability^23^.

In addition, a large number of previous studies have shown that the main components of plants consist of secondary metabolites, and the secondary metabolites in different tissues of plants are significantly different^24^. And the accumulation of plant secondary metabolites produces a stress effect that can change the content of plant secondary metabolism^25^. Therefore, for the expression of genes related to the synthesis of secondary metabolites, the expression profiles may be difference in different tissues or under different stress treatments, and revealing their expression profiles is of great significance for the analysis of the genes. Previous studies in our laboratory showed that the content of oridonin, the main active component of *I. rubescens*, was significantly different in different tissues, and its content was also different in different environments. Therefore, the selection of reference genes in different tissues and under different stress treatments is of great significance for the study of genes in *I. rubescens*. Here, we have for the first time sought to evaluate the stability of 12 candidate reference genes for screening the most suitable reference genes for RT-qPCR data normalization in different sets of *I. rubescens* samples, including different tissues and different stress treatments. The selection of reference genes will provide a foundation for the benefit of future studies of gene expression in *I. rubescens*.

## Results

### Candidate reference genes selection and amplification specificity

**Figure 1:**
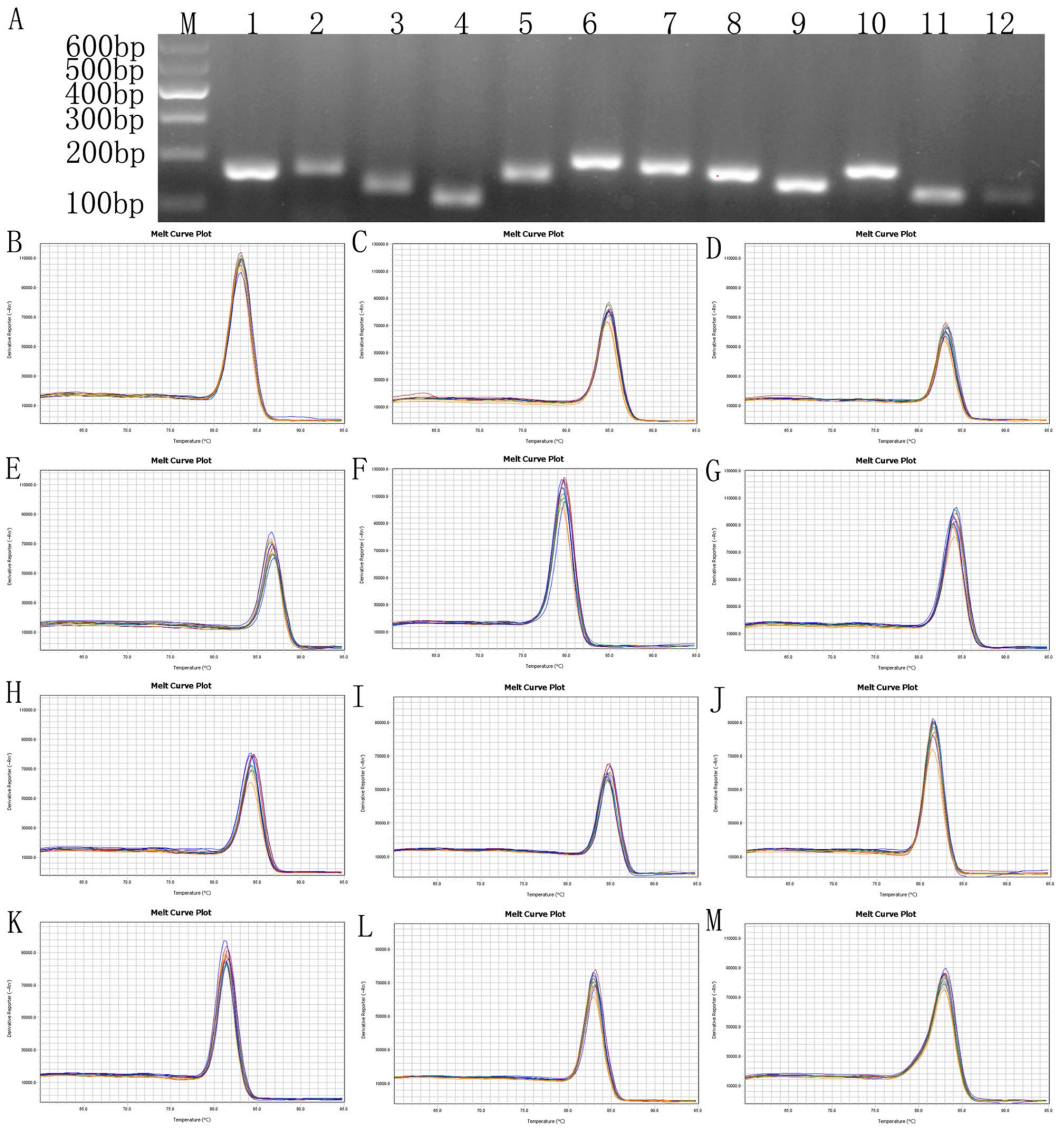
RT-PCR and RT-qPCR amplification specificity. (**A**) Agarose gel (2.0%) electrophoresis indicated the amplification of a single PCR product of the expected size for 12 genes (lines 1–12: *18S*, *TUB*, *ACT*, *Apt*, *HIS*, *UBQ*, *TUA*, *Cycl*, *GAPDH*, *eIF*, *PP2A*, *EF-1α*; (**B**–**M**) *18S, TUB, ACT, Apt, HIS, UBQ, TUA, Cycl, GAPDH, eIF, PP2A, EF-1α*). The melting curves for the 12 genes shows single peaks. M represents a 600 bp DNA marker.

Combined with the transcriptome data of *I. rubescens*, a total of 12 candidate reference genes (*18S*, *TUB*, *ACT*, *Apt*, *HIS*, *UBQ*, *TUA*, *Cycl*, *GAPDH*, *eIF*, *PP2A*, and *EF-1α*) that have been reported to be good potential reference genes in previously published papers were selected for validation as potentially suitable reference genes in *I. rubescens* from different tissues and under different abiotic stresses. The sequences of 12 candidate reference genes were obtained from transcriptome data of *I. rubescens* (Supplementary Table S1), and the primer sequences and descriptions of the candidate reference genes are shown in Table 1. To test the accuracy and specificity of the primers, agarose gel electrophoresis showed a single DNA band in the range of 110–200 bp, which was consistent with the predicted PCR products according to the various primer sets (Fig. 1A, Figure S1). Furthermore, the melting curves by RT-qPCR for the amplified products of all 12 candidate reference genes exhibited a single peak, corresponding to a specific melting temperature which ranged from 79.70 °C (*UBQ*) to 86.67 °C (*HIS*) (Fig. 1B). These two results indicated good specificity of all the primer pairs used in our study. In addition, the PCR efficiency of each candidate reference gene ranged from 1.8900 to 2.0528, among which *18S* had the lowest amplification efficiency, *PP2A* had the highest amplification efficiency, and *TUB* showed the optimal efficiency (Table 1). In conclusion, these results suggest that the designed primers were suitable for RT-qPCR analysis of gene expression.

**Table 1:** Gene description, primer sequences, amplicon length, and PCR efficiency for *I. rubescens* candidate reference genes.

Gene	Gene annotation	arabidopsis ortholog	Primer sequence of forward (5′–3′)	Primer sequence of reward (5′–3′)	Amplicon length (bp)	Tm (°C)	PCR efficiency
*18S*	18S ribosomal RNA	AT3G41768.1	ACCACATCCAAGGAAGGCAG	TCTTCATCGATGCGAGAGCC	156	83.05	1.8900
*TUB*	Tubulin	AT5G23860	GCTCTCACTCACACACCAG	GCACACCACTTCCCAGAACT	166	84.87	1.9936
*ACT*	Actin 7	AT5G09810.1	GCCATGTATGTCGCTATTCAG	TTGCATGGGGAAGAGCGTAAC	133	82.94	2.0360
*Apt*	Adenine phosphoribosyl transferase	AT1G80050.1	GGTGGCGTAACCGTACTCAA	ACACGAATGGTGGAAGCGAT	113	83.09	1.9500
*HIS*	Histone H3.3	AT4G40030.2	ACGATCGTGCGCATTAGGAG	CTTACGGGCAGTTTGCTTGG	160	86.67	2.0260
*UBQ*	Ubiquitin	AT5G20620.1	CTGAGGCTTCGTGGAGGGAT	TGTAGTCTGCAAGAGTCCTT	196	79.70	1.9100
*TUA*	Tubulin alpha chain	AT1G04820	TGCTGAGAAAGCCTACCACG	CTTGGTCTTGATTGTGGCAAC	184	84.09	1.9900
*Cycl*	Cyclophilin	AT4G34960	CCCAGTTCTTCATCTGCACC	GGATCTCAAGCGAGCTGACC	175	84.30	1.8901
*GAPDH*	Glyceraldehyde 3-phosphate dehydrogenase	AT1G13440.1	GGCAAAGTTCTCCCTGCTCT	CCCCTCAGACTCCTCCTTGA	153	84.87	1.9199
*eIF*	Eukaryotic translation initiation factor 5A	AT1G13950.1	ATGTGCCTCATGTGAACCGT	CTGCACAGACACGACGAGAT	182	81.56	1.9107
*PP2A*	Serine/Threonine protein phosphatase 2a	AT1G69960	TGGTGACCGTTTTCAGTGCT	TAGTGTCGGGCTCAATCTGC	135	81.49	2.0528
*EF-1α*	Elongation factor 1-alpha	AT5G60390.1	TAGGTGCGCTCCCTCTACAC	CAATGCTGATGTGAGTCTT	135	83.03	1.9181
*HMGR*	3-hydroxy-3-methylglutaryl-CoA reductase	AT1G76490.1	TCGAAATCCGACAGCGACAT	GGCTTGGCGACTATGGAAGT	75	84.57	1.9871

### Expression profiling of candidate reference genes in *I. rubescens*

**Figure 2:**
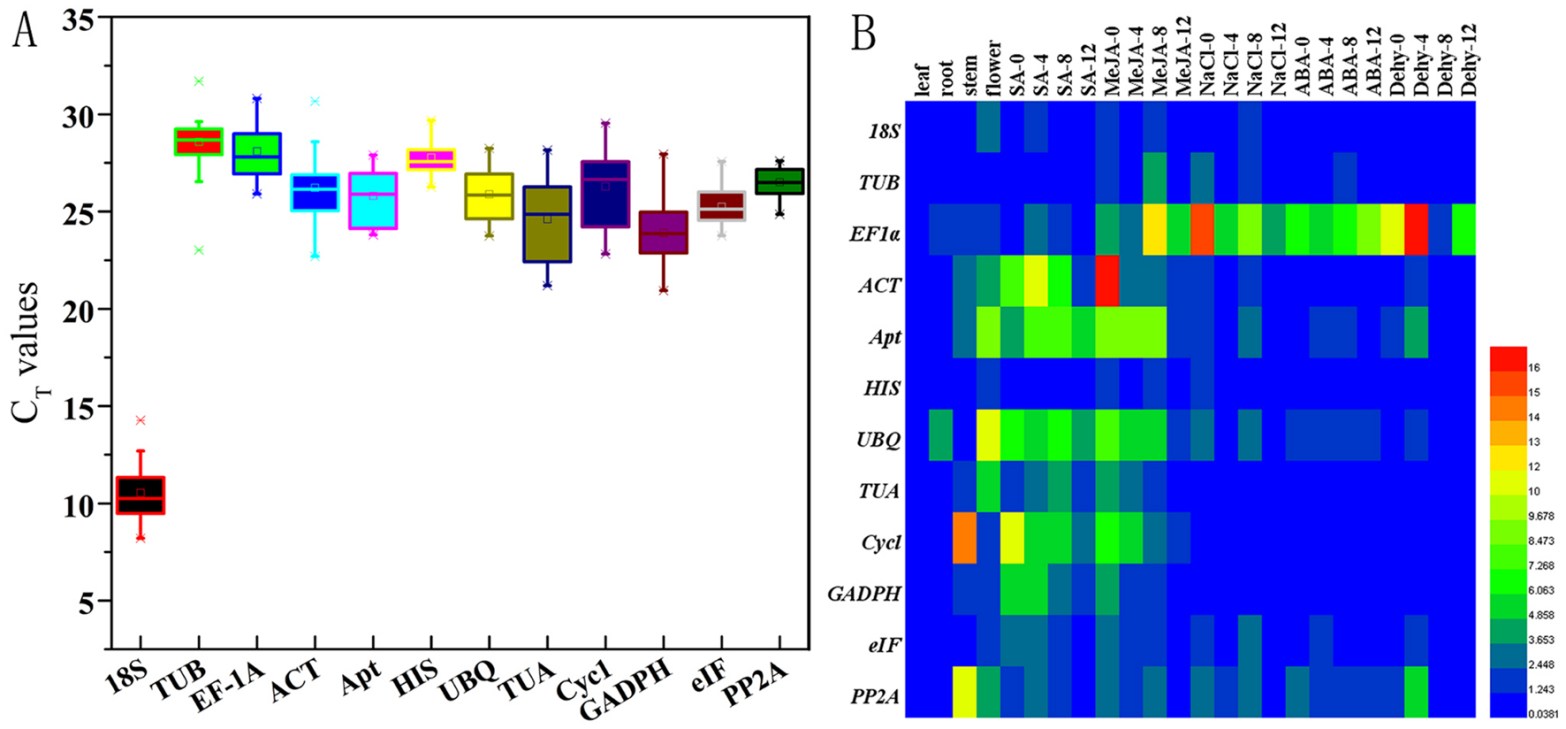
Expression stability of the 12 tested candidate reference genes in all 24 samples. (**A**) Box-plot graphs of Ct-values for each candidate reference gene. The plots highlight the mean (hollow square), median (center line), maximum, and minimum (whiskers, the vertical lines), and 25th and 75th percentiles (boxes) of the data. (**B**) Heat map showing FC fold change values of gene relative expression levels in all 24 samples. The color scale represents the gene relative expression level. Low to high expression is represented over a spectrum from blue to red, respectively.

The threshold cycle (Ct)-values were used to determine the expression levels of 12 tested candidate reference genes in all 24 samples. The Ct values are listed in Supplementary Table S2 and also exhibited as box-plot graphs (Fig. 2A). The results showed that *18S* were the highest expressed genes, with the lowest mean Ct-values of 10.55 in the different samples of *I. rubescens*. The mean Ct-values of others candidate reference gene ranged from 23.90 to 28.11, among which *TUB* showed the highest Ct-values of 28.81, and was the lowest expressed gene in all samples tested (Fig. 2A). Obviously, although each candidate reference gene showed expression difference, *18S* displayed high expression. This suggests that *18S*, a commonly used traditional reference gene, can be selected as an internal reference gene for genes with high expression. Furthermore, the 2^−∆Ct^ method was used to analyze the expression stability of 12 tested candidate reference genes in all 24 samples. In this study, the fold change of not treated leaf sample was selected as a calibrator. The heatmap directly revealed the stability of gene expression in all samples (Fig. 2B). The expression levels of *18S*, *TUB*, *HIS*, and *eIF* were stable, while *EF-1α*, *TUA*, and *Cycl* showed large variations in expression levels.

### Expression stability analysis of candidate reference genes in *I. rubescens*

**Figure 3:**
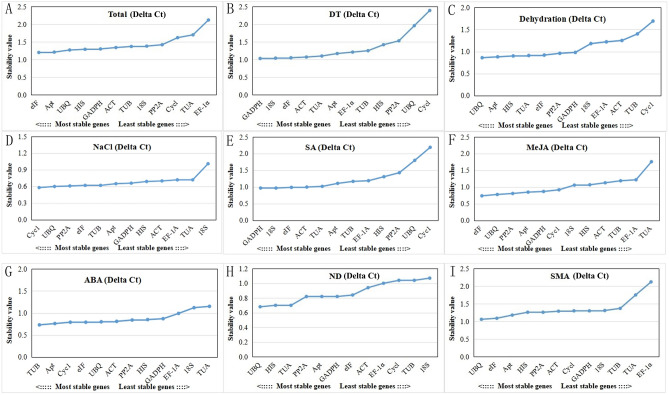
Candidate reference gene ranking based on ∆Ct method. (**A**) Total: the set of total samples. (**B**) DT: the set with the samples of different tissues. (**C**) Dehydration treatment samples. (**D**) NaCl treatment samples. (**E**) SA treatment samples. (**F**) MeJA treatment samples. (**G**) ABA treatment samples. (**H**) ND: the set with the samples of NaCl and dehydration treatments. (**I**) SMA: the set with the samples of SA, MeJA, and ABA treatments.

In this study, four major computational algorithms were used to screen suitable candidate reference genes: the comparative ∆Ct method, BestKeeper, Normfinder, and geNorm. In the method of comparative ∆Ct, the stability of the candidate reference genes was assessed by the index of the mean standard deviation (STDEV). A low STDEV value of a candidate reference gene indicated that the gene was expressed stably in different samples. According to the results of this method, *eIF* was the most stable reference gene showing the lowest STDEV for all the samples and methyl jasmonate (MeJA) treatment of *I. rubescens* (Fig. 3A,F). *GADPH* was the most stable reference gene for the different tissues (DT) and salicylic acid (SA) treatment samples (Fig. 3B,E). *Cycl* and *TUB* exhibited the most stable reference gene for the NaCl and abscisic acid (ABA) treatment samples, respectively (Fig. 3D,G). *UBQ* was the most stable reference gene for dehydration treatment samples, ND set (combine NaCl and dehydration treatment samples) and SMA set (combine SA, MeJA, and ABA treatment samples) (Fig. 3C,H,I).

**Figure 4:**
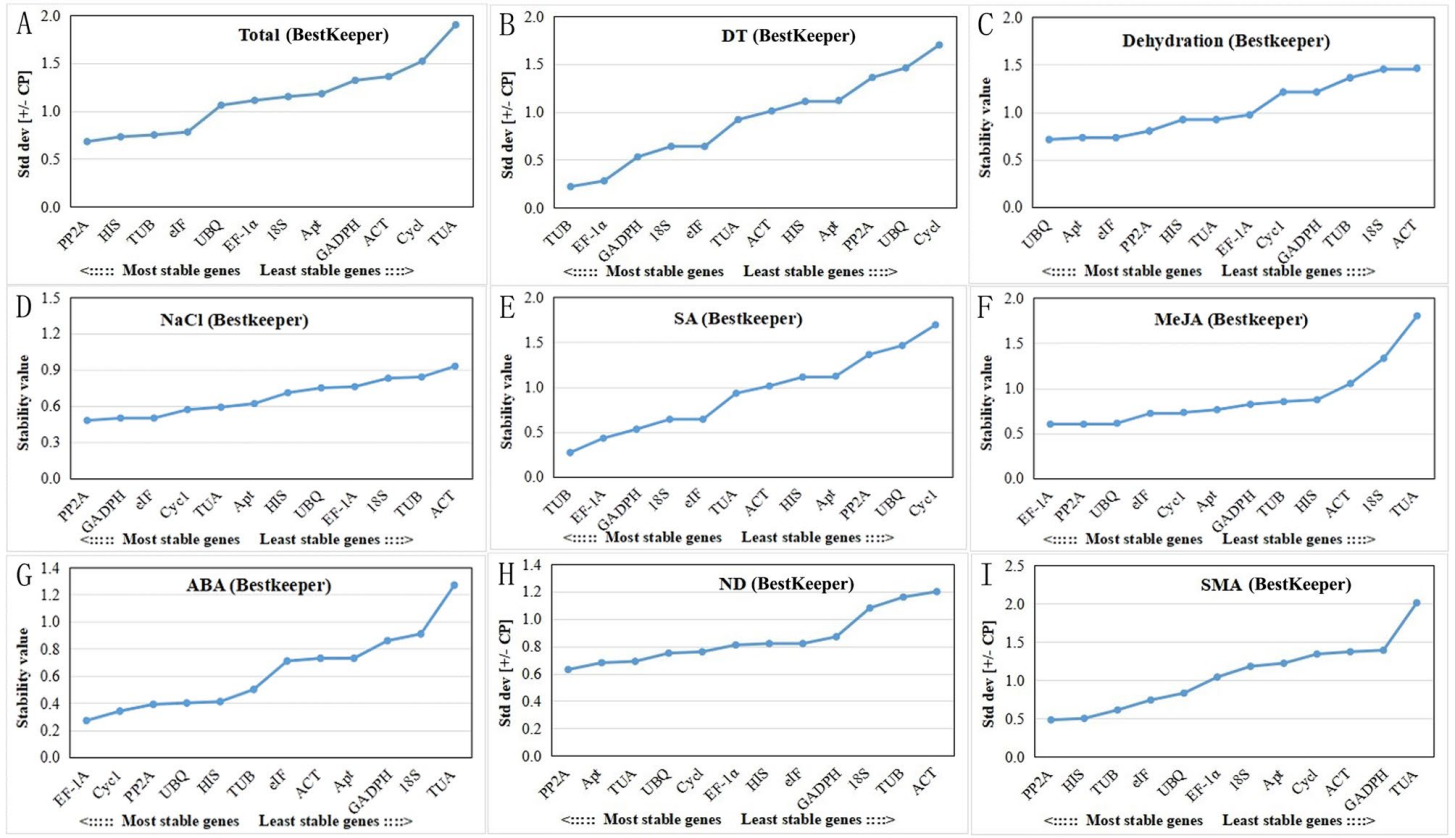
BestKeeper-based expression stability evaluation of 12 candidate reference genes. (**A**) Total: the set of total samples. (**B**) DT: the set with the samples of different tissues. (**C**) Dehydration treatment samples. (**D**) NaCl treatment samples. (**E**) SA treatment samples. (**F**) MeJA treatment samples. (**G**) ABA treatment samples. (**H**) ND: the set with the samples of NaCl and dehydration treatments. (**I**) SMA: the set with the samples of SA, MeJA, and ABA treatments.

The BestKeeper software tool is an Excel-based tool that uses the values of the standard deviation (STDEV) and the coefficient of variation (CV) of each candidate reference gene to estimate the gene expression stability in different samples. According to the standard values determined by BestKeeper, reference genes with STDEV > 1 are considered unstable and should be avoided^26^. In this study, there were four genes with STDEV < 1, including *PP2A*, *HIS*, *TUB*, and *eIF*, which showed that these genes possessed stable expression in total samples with SD-values of 0.68, 0.73, 0.75, and 0.78, respectively. The most stable gene in total samples was *PP2A*, followed by *HIS*, *TUB*, and *eIF*, while the least stable gene was *TUA* (Fig. 4A). For the DT and SA samples, *TUB* was deemed the most stable gene (Fig. 4B,E). *EF-1α* was determined to be the most stable gene both in the MeJA samples and ABA samples (Fig. 4F,G). *UBQ* was deemed the most stable gene in the dehydration samples (Fig. 4C). *PP2A* was determined to be the most stable gene in the NaCl, ND and SMA samples by the BestKeeper software (Fig. 4D,H,I).

**Figure 5:**
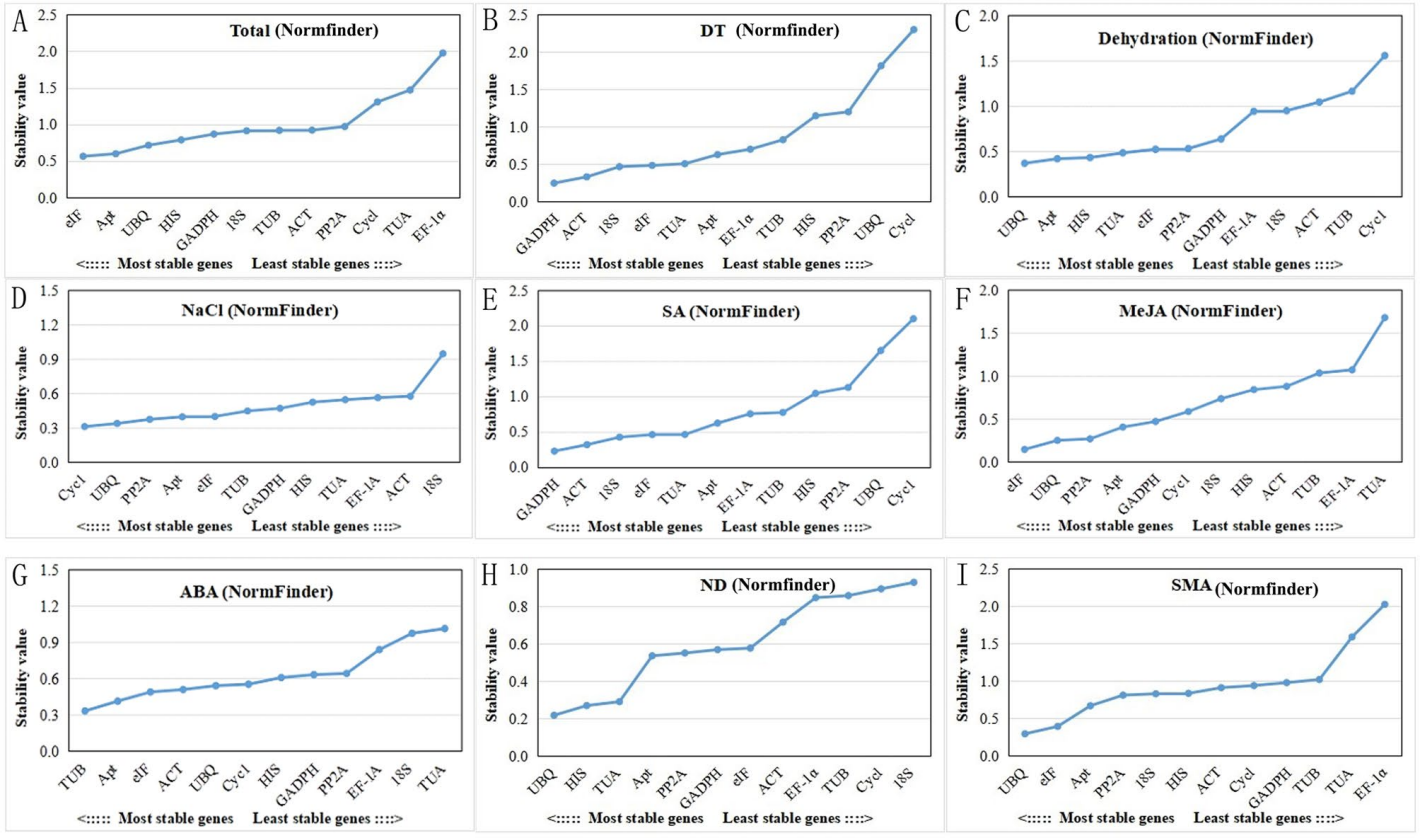
NormFinder-based expression stability evaluation of 12 candidate reference genes. (**A**) Total: the set of total samples. (**B**) DT: the set with the samples of different tissues. (**C**) Dehydration treatment samples. (**D**) NaCl treatment samples. (**E**) SA treatment samples. (**F**) MeJA treatment samples. (**G**) ABA treatment samples. (**H**) ND: the set with the samples of NaCl and dehydration treatments. (**I**) SMA: the set with the samples of SA, MeJA, and ABA treatments.

Normfinder is another algorithm for identifying the optimal reference gene among a set of candidates, and this algorithm can also calculate the variation between subgroups of the total sample set^27^. According the result of Normfinder, as the stability values (SV) decreased, the stability of the candidate reference genes increased. In the total samples of *I. rubescens*, *eIF* ranked first with an SV of 0.0.564, followed by *Apt* and *UBQ* with SV of 0.599 and 0.715, while, *EF-1α* ranked last with an SV of 1.974 (Fig. 5A). *GADPH* was the most suitable candidate genes for normalization with an SV of 0.245 in the DT samples (Fig. 5B). *UBQ*, *Cycl*, *GADPH*, *eIF*, *TUB*, and *TUB* ranked first and deemed as the most stable gene in the dehydration, NaCl, SA, MeJA, and ABA samples, respectively (Fig. 5C–G). In addition, in the composite group of ND samples and SMA samples, *UBQ* ranked first with an SV of 0.292, and 0.217, respectively (Fig. 5H,I). The results of Normfinder were similar when compared with the results of the ∆Ct method.

GeNorm, an applet for Microsoft Excel, was used to calculate the gene expression stability measure M (M-value) for a reference gene as the average pairwise variation V for that gene with all other tested reference genes^28^. According to the algorithm used by GeNorm, the two most stable reference genes were determined based on the M-value. The M-value is inversely related to stability of the candidate reference genes, the lower the M-value is, the higher the stability will be, conversely, the higher the M-value is, the lower the stability will be. In our analysis, the most stable genes were *ACT* and *GADPH* with an M-value of 0.619 in Total samples, followed by *Apt* and *UBQ* with the M-values of 0.856 and 0.926, respectively. The most unstable gene was *EF-1α* with an M-value of 1.447. In DT and SA samples, *18S* and *eIF* had an M-value of 0.2826 and 0.297, respectively, displaying the most stable expression. For the MeJA, ABA, Dehydration, and NaCl samples, *UBQ* and *eIF*, *Cyc1* and *PP2A*, *Apt* and *eIF*, *TUB* and *ACT* were deemed as the most stable expression genes with an M-value of 0.288, 0.213, 0.414, and 0.191, respectively. In addition, *HIS* and *UBQ* were the most suitable candidate genes for normalization with an M-value of 0.2465 in ND samples, whereas *ACT* and *GADPH* were the most stable genes with an M-value of 0.5530 in SMA samples (Table 2).

**Table 2:** Expression stability M-value of 12 candidate reference genes calculated by geNorm.

Stability rank	Total		DT		SA		MeJA		ABA		Dehydration		NaCl		ND		SMA	
	Gene	Values	Gene	Values	Gene	Values	Gene	Values	Gene	Values	Gene	Values	Gene	Values	Gene	Values	Gene	Values
1	ACT/GADPH	0.6187	18S/ eIF	0.2826	18S/eIF	0.297	UBQ/eIF	0.288	Cyc1/PP2A	0.213	Apt/ eIF	0.414	TUB/ACT	0.191	HIS/UBQ	0.2465	ACT/GADPH	0.553
2	Apt	0.8555	GADPH	0.526	GADPH	0.496	Apt	0.342	UBQ	0.371	UBQ	0.466	Cyc1	0.379	Apt	0.4579	Cycl	0.6911
3	UBQ	0.9261	TUA	0.6244	TUA	0.58	PP2A	0.389	HIS	0.446	HIS	0.522	PP2A	0.405	PP2A	0.5109	UBQ	0.7893
4	eIF	0.9903	ACT	0.6961	ACT	0.645	Cyc1	0.439	EF-1α	0.495	TUA	0.549	UBQ	0.431	TUA	0.547	Apt	0.8151
5	HIS	1.0642	Apt	0.7242	Apt	0.678	GADPH	0.478	TUB	0.547	PP2A	0.593	HIS	0.46	eIF	0.5977	eIF	0.8656
6	18S	1.1069	EF-1α	0.7949	TUB	0.752	ACT	0.585	ACT	0.621	GADPH	0.652	EF-1α	0.479	GADPH	0.6296	18S	0.9476
7	TUB	1.1385	TUB	0.8317	EF-1α	0.802	18S	0.691	Apt	0.683	18S	0.746	Apt	0.524	EF-1α	0.704	PP2A	1.0321
8	PP2A	1.1767	HIS	0.8929	HIS	0.852	HIS	0.769	eIF	0.722	ACT	0.809	eIF	0.566	Cycl	0.7464	HIS	1.072
9	TUA	1.2512	PP2A	1.0026	PP2A	0.953	TUB	0.841	GADPH	0.753	EF-1α	0.888	GADPH	0.592	ACT	0.7871	TUB	1.1157
10	Cycl	1.3097	UBQ	1.1452	UBQ	1.077	EF-1α	0.888	18S	0.819	TUB	0.978	TUA	0.617	TUB	0.8313	TUA	1.2041
11	EF-1α	1.4472	Cycl	1.3525	Cyc1	1.263	TUA	1.034	TUA	0.874	Cyc1	1.096	18S	0.683	18S	0.8707	EF-1α	1.3568

GeNorm was also used to revealed the optimal number of reference genes for normalization^21^. The pairwise variation between the normalization factors NF_n_ and NF_n+1_ of two sequences (V_n/n+1_) was calculated. If V_n/n+1_ was more than 0.15, the number of genes used in normalization was n + 1, until V_n/n+1_ was less than 0.15, the number of genes used in normalization was n. In this study, the value of n was different in the four sample sets. For the total samples, six reference genes were needed for normalization since the V_6/7_-value (0.1451) was inferior to the 0.15 cut-off level. For ND samples, three reference genes were required for accurate RT-qPCR data normalization since the V_3/4_-value (0.1236) was < 0.15. And in the DT and SMA samples, four reference genes were required since the V_4/5_-values were 0.1423 and 0.1406, respectively (Table 3). In addition, for the remaining samples of dehydration, NaCl, SA, MeJA, and ABA samples, only two reference genes were required since the V_2/3_-values were all inferior to the 0.15 cut-off level.

**Table 3:** Pairwise variation by geNorm determine the optimal number of reference genes.

Pairwise variations	Total (values)	DT (values)	Dehydration (values)	NaCl (values)	SA (values)	MeJA (values)	ABA (values)	ND (values)	SMA (values)
V_2/3_	0.3034	0.2120	0.0744	0.0726	0.1276	0.1244	0.1111	0.1846	0.2361
V_3/4_	0.2200	0.1640	0.0772	0.0560	0.0738	0.0929	0.0770	0.1236	0.2005
V_4/5_	0.1861	0.1423	0.0767	0.0507	0.0910	0.1016	0.0968	0.1021	0.1406
V_5/6_	0.1661	0.1135	0.0729	0.0721	0.0652	0.0911	0.0940	0.1057	0.1379
V_6/7_	0.1451	0.1231	0.1076	0.0613	0.0570	0.1288	0.1124	0.0873	0.1461
V_7/8_	0.1289	0.1044	0.1053	0.0754	0.0636	0.1284	0.1037	0.1078	0.1443
V_8/9_	0.1213	0.1109	0.0943	0.0771	0.0737	0.1142	0.0911	0.0870	0.1168
V_9/10_	0.1338	0.1352	0.0849	0.0651	0.0769	0.1133	0.0838	0.0825	0.1138
V_10/11_	0.1229	0.1510	0.1027	0.0561	0.0871	0.0976	0.0915	0.0811	0.1339
V_11/12_	0.1661	0.1928	0.1111	0.0831	0.0788	0.1556	0.0903	0.0787	0.1699

### Comprehensive ranking of the reference genes by RankAggreg

**Figure 6:**
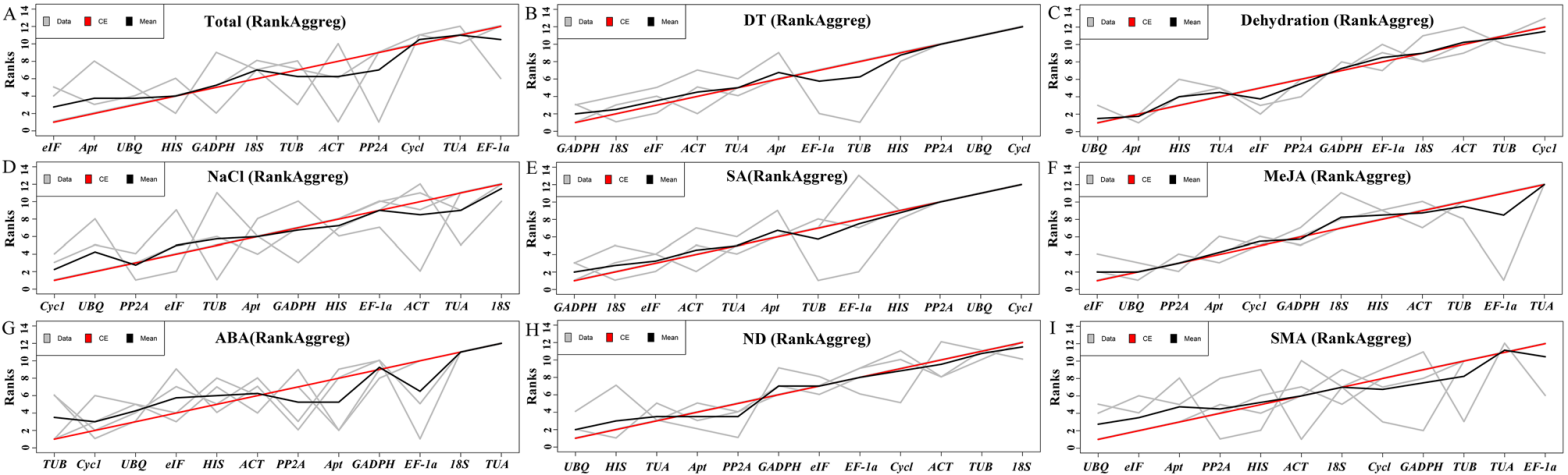
The comprehensive ranking list of 12 genes by RankAggreg. The gray lines show the ranking lists of ∆Ct, NormFinder, BestKeeper and geNorm. The black line indicates the mean rank position of each gene. The red line indicates the result of RankAggreg with the Monte Carlo algorithm and Spearman footrule distances. (**A**) Total: the set of total samples. (**B**) DT: the set with the samples of different tissues. (**C**) Dehydration treatment samples. (**D**) NaCl treatment samples. (**E**) SA treatment samples. (**F**) MeJA treatment samples. (**G**) ABA treatment samples. (**H**) ND: the set with the samples of NaCl and dehydration treatments. (**I**) SMA: the set with the samples of SA, MeJA, and ABA treatments.

The ∆Ct method, BestKeeper, Normfinder, and geNorm exhibited different ranking results in their stability analysis of candidate reference gene expression in this study. For the total samples, the comparative ∆Ct method (Fig. 2) and NormFinder (Fig. 4) analysis ranked *eIF* as the most stable, while BestKeeper suggested *PP2A* was the most appropriate reference gene (Fig. 3), and GeNorm analysis showed *ACT* and *GADPH* to be the two most stable genes (Table 2). The results of the other sets showed similar variable results. Thus, a comprehensive tool RankAggreg, known as a non-weighted unsupervised rank aggregation method, was used to evaluate the comprehensive ranking list of all genes based on the merged results of the ∆Ct, Bestkeeper, NormFinder and geNorm methods^23^. The results analysed by RankAggreg indicate that the most suitable reference genes tested for normalization in Total samples were *eIF*, *Apt*, and *UBQ*. In DT set, the best three candidate reference genes were *GADPH*, *18S*, and *eIF*. *UBQ, Apt,* and *HIS*; *Cycl, UBQ,* and *PP2A*; *GADPH, 18S,* and *eIF*; *eIF, UBQ,* and *PP2A; TUB, Cycl,* and *UBQ*; were the best three candidate reference genes for the samples of Dehydration, NaCl, SA, MeJA, and ABA treatment, respectively. While in ND set, *UBQ*, *HIS*, and *TUA* were the three appropriate candidate reference genes. In addition, *UBQ*, *eIF* and *Apt* were top-ranked in the SMA set (Fig. 6).

### Validation of the selected reference genes

**Figure 7:**
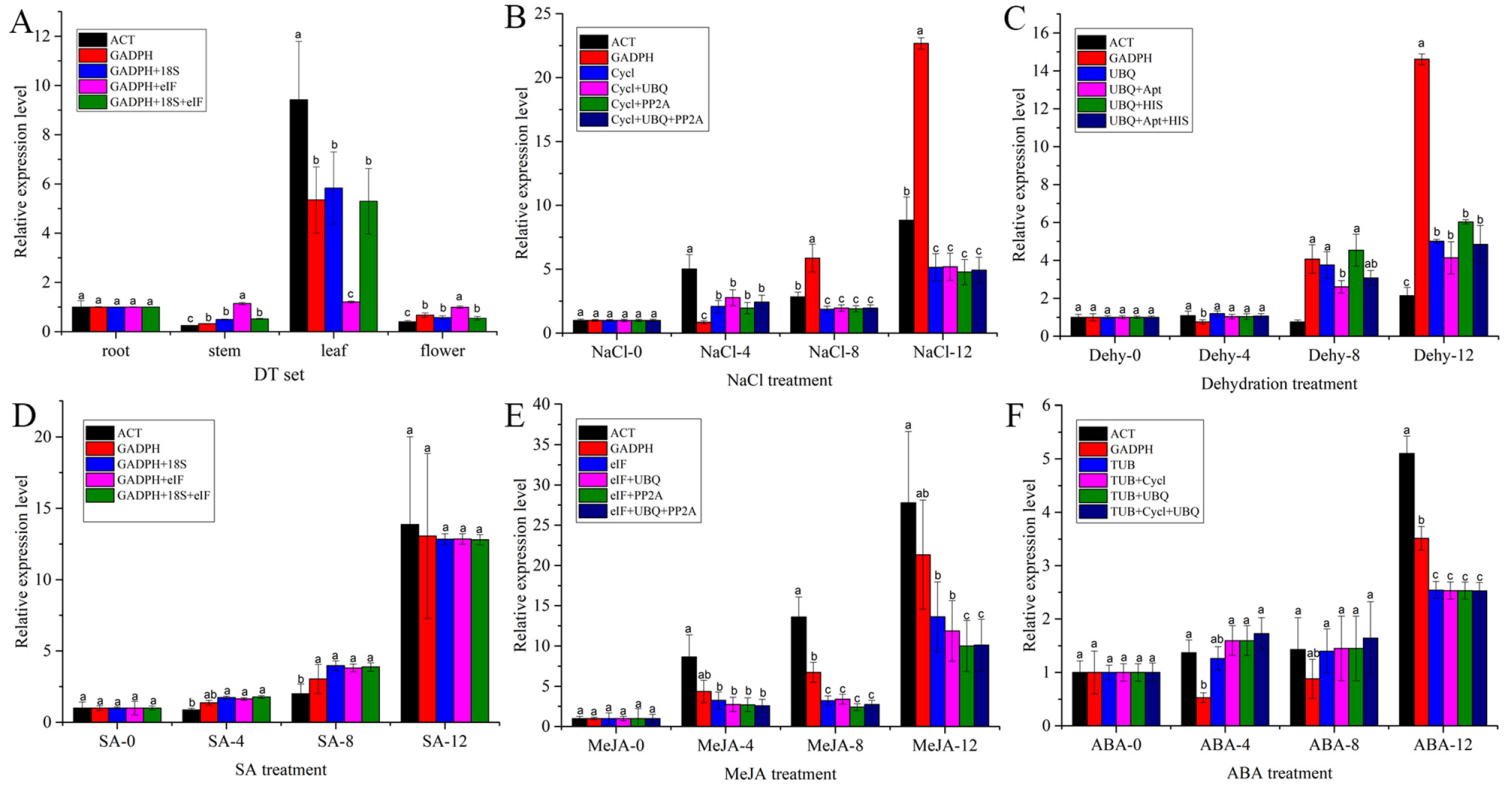
Relative expression levels of *HMGR* in *I. rubscences*. The expression of *HMGR* in *I. rubscences* in different tissues (**A**), under NaCl (**B**), dehydration (**C**), SA (**D**), MeJA (**E**) and ABA (**F**) treatments. Genes were normalized to *ACT* or *GADPH* or a combination of the two or three top ranked genes defined by the RefFinder and RankAggreg analysis. All values are means ± SD (n = 3). Different letters on the bar indicate a significant difference at p < 0.05.

Previously, only one *ACT*^7^ or *GADPH*^29^ gene was used as a reference gene in *I. rubscences* for data normalization. However, the results of our study show that *ACT* appears to be not most stably expressed reference gene in *I. rubscences*, especially in different tissues. *GADPH* appears stably expressed in different tissues, but not under other conditions (Fig. 3). Therefore, in order to validate the reliability of the three most stable candidate reference genes determined by RankAggreg (Fig. 6), all were either individually or in combination used as internal controls to analyze the expression pattern of 3-hydroxy-3-methylglutaryl-CoA reductase (*HMGR,* accession number: MK344323.1), the first rate-limiting enzyme of mavalonic acid pathway, in the different samples of *I. rubscences*. The results were then compared to *ACT* or *GADPH* for data normalization. As shown in Fig. 7, the results showed the same expression patterns when using the combination of the two or three most stable genes or *ACT* or *GADPH* reference genes to normalize RT-qPCR data. However, the accumulation of *HMGR* transcripts was under- or over-estimated when the data were normalized using *ACT* or *GAPDH* as internal controls alone (Fig. 7). This result suggests that the determination of gene expression levels was obviously affected when unstable reference genes were used for normalization, and two or more reference genes contributed to the reliability of the results. Taken together, considering the cost and operation process, we recommend using *GAPDH* or combined with *18S* in different *I. rubscences* tissues to normalize RT-qPCR data of gene expression. *UBQ* alone*,* or combined with *Apt,* and *HIS*; *Cycl* alone*,* or combined with *UBQ,* and *PP2A*; *GADPH* alone*,* or combined with *18S,* and *eIF*; *eIF* alone*,* or combined with *UBQ,* and *PP2A; TUB* alone*,* or combined with *Cycl,* and *UBQ*; to normalize RT-qPCR data of gene expression. for the samples under Dehydration, NaCl, SA, MeJA, and ABA treatment, respectively.

## Discussion

RT-qPCR is an accurate and sensitive technology used for the detection of gene expression levels. To obtain successful gene expression by RT-qPCR, it is crucial to select an appropriate reference gene. In the past few years, with the increase in research on medicinal plant genes, studies aimed at selecting appropriate reference genes as internal controls for RT-qPCR analysis have been reported in many medicinal plant species^14–16^. Furthermore, more evidence shows that the expression stability of reference genes may be altered across different tissues or under different experimental conditions. In *Panax ginseng*, *GAPDH*/*30S RPS20*, *CYP/60S RPL13*, and *CYP/QCR* were the optimum pair of reference genes in the roots, stems, and leaves^30^. In *Metasequoia*, *ACT2*, *HIS*, and *TATA* were stably expressed in different tissues. While *EF-1α*, *HIS*, and *TATA* were optimal for abscisic acid-response^21^. In the *Populus euphratica*, the genes selected as optimal reference genes in different treatments were varied: *RPL17* in ABA; *EF-1α* in cold; *HIS* in dehydration; *GII*α in drought and short-duration salt; and *TUB* in long-duration salt^31^. However, as for *I. rubscences*, *ACT*^7^ or *GADPH*^29^ was frequently used as the internal controls in RT-qPCR without any form of stable reference gene examination, and this results in a lack of a certain degree of rigor. Thus, it was necessary to investigate the expression stability of candidate reference genes under different experimental conditions prior to using them for RT-qPCR data normalization.

In our work, 12 genes commonly used as internal reference genes in previous articles were selected as candidate reference genes, and six statistical algorithms (ΔCt, Bestkeeper, NormFinder, geNorm, RefFinder, and RankAggreg) were used in gene expression studies of *I. rubscences* from different tissues or under different abiotic stresses (Figure S2). We analyzed the candidate reference genes of each group under different treatment separately. Furthermore, in order to avoid the complexity of the experiment caused by too much subdivision of the six sets, we also set up three groups, including different tissue samples (DT set), NaCl and dehydration treatment samples (ND set), SA, MeJA, and ABA treatment samples (SMA set), so as to facilitate the precise application of internal reference genes under different environments conditions. In addition, in order to comprehensively compare the expression levels of genes in different samples, we also carried out screening and analysis of internal reference genes for all samples. As shown in Fig. 6, Comprehensive Ranking of the Reference Genes by RankAggreg, which used the output data of ∆Ct, BestKeeper, NormFinder, and GeNorm methods to determine suitable reference genes in different sets, and the results indicate *eIF*, *Apt*, and *UBQ* were optimal for the whole sample analysis. *GADPH*, *18S*, and *eIF* were stably expressed in different tissues. *UBQ, Apt,* and *HIS*; *Cycl, UBQ,* and *PP2A*; *GADPH, 18S,* and *eIF*; *eIF, UBQ,* and *PP2A; TUB, Cycl,* and *UBQ*; were the best three candidate reference genes for the samples of Dehydration, NaCl, SA, MeJA, and ABA treatment, respectively. In addition, *UBQ*, *HIS*, and *PP2A* were appropriate reference genes in the combination of NaCl or in the dehydration treatment, while *UBQ*, *eIF* and *PP2A* were optimal for the combination of SA, MeJA or ABA treatment. Therefore, the overall analysis results provide a basis for the appropriate selection of internal reference genes to study gene expression levels under different treatments in samples of *I. rubscences*.

The expression values of stable internal reference genes are not constant in different samples. As shown in Fig. 2, each candidate reference gene showed different Ct ranges in all samples, and the stability of each gene in different samples was clearly different. As a single software package may introduce bias, ∆Ct and geNorm, BestKeeper, and NormFinder were used for evaluation of candidate reference genes^32^. It can be seen that the different methods mentioned above yielded different ranking patterns. For the total samples, *eIF*, *Apt*, and *UBQ* were suitable genes by the ∆Ct (Fig. 3) and NormFinder (Fig. 5) methods, *PP2A*, while *HIS* and *TUB* were more stable than others by BestKeeper (Fig. 4), and *ACT* and *GADPH* were the most stable genes based on geNorm (Table 2). For the analysis of other sets, there were similar phenomena; the results of different calculation methods showed different stability ranking lists of genes. The above results show that a single calculation method may introduce bias. Thus, RankAggeg, a comprehensive evaluation tools, were used to determine the final comprehensive ranking list of all genes^14^. As shown in Fig. 6, *UBQ* was relatively stable, especially in the ND set and SMA set, while it was less stable in the DT set. This inconsistency might be due to the different tissues used. *UBQ* is one of the most commonly used reference gene in many species, such as in NaCl-treated and ABA-treated *Platycladus orientalis*^33^, during somatic embryogenesis in longan tree^34^, and across all samples of *Gossypium hirsutum*^35^. However, our study demonstrated that *UBQ* was not stable in different tissues when compared with other reference genes, and *GAPDH* was the most stable in different tissues of *I. rubscences* (Figs. 3B, 5B). These results show that the stability of different internal reference genes is also affected by species. In *Metasequoia*, *UBQ* was also not stable in different tissues^21^. A traditional reference gene *GAPDH* was also one of the most used reference gene in many species. In *Eremosparton songoricum*, *EsGAPDH* was one of the most stable genes across multiple adult tissue samples^36^, as the same results were seen in *Galium aparine* with different tissues^8^, and at different developmental stages of *Jatropha curcas*^37^. These results demonstrate that different reference genes exhibit variable gene expression patterns in different species.

In most gene expression studies, only one reference gene was adopted for RT-qPCR data normalization. However, the use of one reference gene was more than likely to result in bias^38^. Therefore, the use of multiple reference genes has been adopted in more and more studies^39^. Additionally, some journals require at least two validated reference genes for normalization, such as Journal of Experiment Botany. In our study, GeNorm was used to select the optimal number of reference genes for normalization: for total samples, DT, ND and SMA sets, six, four, three and four reference genes were the optimal number, respectively (Table 3). In addition, to validate the selected reference genes, the three most stable genes were used as internal controls to normalize the RT-qPCR data from *HMGR* and were compared with *ACT* and *GADPH* normalization (Fig. 7). The results showed that the expression levels of the target gene *HMGR* were significantly changed when different reference genes were used for normalization, leading to unreliable experimental results. Our results support that a combination of multiple reference genes is preferred rather than a single internal reference gene. Similar results were also reported in other plant species using multiple reference gene normalizers^14^. Above all, the results acquired in this study will improve the accuracy of quantification of target gene expression with RT-qPCR analysis in *I. rubscences* and support the importance of selection of suitable reference genes for future gene expression studies in this species.

## Materials and methods

### Plant material treatment and collection

The seeds of *I. rubescens* were collected from Jiyuan, an experimental base of our affiliated institute, Henan province. At the reproductive stage of *I. rubescens* grown in an illumination incubator for 6 months after germination with relative humidity of 70% under a 16 h/8 h light/dark photoperiod (120 µmol m^−2^ s^−1^) at 24–26 °C, different tissues (DT set) of roots, stems, leaves, and flowers were collected. At the vegetative stage 2 months after germination, the *I. rubescens* seedlings were treated with different abiotic stresses. For the simulated salt stress treatment, the uniformly developed seedlings of *I. rubescens* were fully watered using a solution of 200 mM NaCl, and the leaves between three and six nodes were harvested at 0, 4, 8, and 12 h after treatment. For the dehydration stress treatment, the uniformly developed seedlings of *I. rubescens* were carefully removed from the soil and washed with tap water. The seedlings were then placed on filter paper and left on the lab bench, and the leaves were collected at 0, 2, 4, and 8 h after the initiation of the dehydration treatment. We designated these two abiotic stress samples as the ND set. For the salicylic acid (SA), methyl jasmonate (MeJA), and abscisic acid (ABA) treatment (SMA set), 200 µmol SA, 200 µmol MeJA, and 300 µmol ABA were each evenly sprayed on the leaves of *I. rubescens*. The leaves between three and six nodes were harvested at 0, 4, 8, and 12 h after treatment. All collected samples were frozen in liquid nitrogen immediately and then preserved at − 80 °C for later analysis.

### Selection of candidate reference genes, primer design, and RT-qPCR

To identify potential candidate reference genes of *I. rubescens*, homologous genes of *Arabidopsis* commonly used as internal controls for gene expression were selected. The assembled transcriptome database of *I. rubescens* was searched using Blastx with an E = 10^−50^ by the NCBI local blast of Bioedit 7.1.9^40^. Twelve candidates reference genes (*18S*, *TUB*, *ACT*, *Apt*, *HIS*, *UBQ*, *TUA*, *Cycl*, *GAPDH*, *eIF*, *PP2A*, *EF-1α*) were selected to investigate the effect of the choice of reference genes on RT-qPCR normalization. Specific primers pairs were designed for each candidate reference gene by the Primer designing tool from NCBI (https://www.ncbi.nlm.nih.gov/tools/primer-blast/), with PCR product size of 70–250 bp, primer melting temperatures of 57–63 °C, and screening of the most suitable primer pair by DNAMEN7.0 (Lynnon Biosoft, Quebec, Canada).

### Total RNA isolation, cDNA synthesis, RT-qPCR, and amplification efficiency

Total RNA of all different samples from *I. rubescens* was extracted using the EASYspin Plus Plant RNA Kit (AidLab, Beijing, China) following the manufacturer’s instructions. RNA quantity and purity were confirmed by measuring the optical density ratio OD_260_/OD_280_ (1.8–2.2) and OD_260_/OD_230_ absorption ratio (1.7–2.0) using a NanoDrop 2000 spectrophotometer (Thermo Fisher Scientific, Waltham, MA, USA). The integrity of the total RNA was checked on 1.0% agarose gels, which showed only two rRNA subunits (28S and 18S) with no indication of RNA degradation. After adjusting each of the RNA samples to the same concentration, 800 ng RNA samples were prepared for the synthesis of cDNA strands. cDNA synthesis use random hexamers was performed following the kit of EasyScript All-in-One First-Strand cDNA Synthesis SuperMix for qPCR (One-Step gDNA Removal) (Transgen, Beijing, China) in accordance with the manufacturer’s instructions.

In this study, the RT-qPCR experimental steps were performed according to a previously published protocol^41^ and MIQE guidelines^42^. RT-qPCR amplification was performed using TransStart Top Green qPCR SuperMix (Transgen, Beijing, China) in accordance with the manufacturer’s instructions and was carried out on the ABI QuantStudio 5 (ABI, America). A 20 µL total reaction mixture contained 100 ng cDNA as a template, 10 µL 2 × TransStart Top Green qPCR SuperMix, 0.4 µL 50 × Passive Reference Dye II, 0.4 µL each specific forward and reverse primer (10 µM), and Nuclease-free water. The reaction condition was as follows: 94 °C for 30 s; 45 cycles of 94 °C for 5 s, and 60 °C for 30 s. For the PCR efficiency analysis, equal amounts of each cDNA sample were pooled. Standard curves were then determined by using 2.5×, 5×, 10×, 100× serial dilutions of pooled cDNA as a template for producing a linear regression model which was calculated by the equation: E = 10^(−1/slope)^^26^. Each assay included three technical replicates.

### Analysis of stability of candidate reference genes

The Ct-values of the 12 reference genes in 24 different samples of *I. rubescens* were used to analyze the gene expression stability levels generated by the QuantStudio 5 software. Four algorithms were used to evaluate the stability of the eight candidate reference genes: the comparative ∆Ct method^17^, NormFinder^19^, BestKeeper^20^, geNorm^18^, and RankAggreg (https://cran.r-project.org/web/packages/RankAggreg/index.html). The other statistical analyses software used in this study included SPSS v 19.0 (https://www.ibm.com/products/spss-statistics), Origin8.0 (http://www.origin8.com/), and Heml 1.0 (http://hemi.biocuckoo.org/)^43^.

### Validation of reference gene stability

To validate the reliability of the selected reference genes and to determine if the normalization of the expression data for a gene of interest is affected by the use of different reference genes, the relative expression profiles of 3-hydroxy-3-methylglutaryl-CoA reductase (*HMGR,* accession number: MK344323.1), the first rate-limiting enzyme of mavalonic acid pathway, in samples of *I. rubescens* different tissues, and leaves under different stresses were analyzed and normalized to the most stable reference genes identified by RankAggreg. By comparison, *ACT*^7^ or *GADPH*^29^ reference genes were also used. The relative expression of *HMGR* was calculated using the 2^-∆∆Ct^ method^44^. Three technical replicates were performed for each biological sample. Values are reported as the mean ± SD from three independent experiments. Statistical analyses were performed using variance (ANOVA) followed by Duncan’s new multiple range tests with SPSS version 19.0 (SPSS, Chicago, IL, USA). Different letters represented significant differences at p < 0.05.

### Ethical approval

We compliance of field studies for collection of plant or seed specimens with relevant guidelines by the IUCN Policy Statement on Research Involving Species at Risk of Extinction and the Convention on the Trade in Endangered Species of Wild Fauna and Flora. And we does not contain any studies with human or animal subjects.

## Conclusions

In this study, we conducted the first comprehensive analysis of the selection of stable reference genes for RT-qPCR analysis of target gene expression in different tissues or under different treatments of *I. rubscences.* The combination of *GAPDH*, *18S* and *eIF* was suitable for gene quantification in different tissues of *I. rubscences*.*UBQ, Apt,* and *HIS*; *Cycl, UBQ,* and *PP2A*; *GADPH, 18S,* and *eIF*; *eIF, UBQ,* and *PP2A; TUB, Cycl,* and *UBQ*; were the best three candidate reference genes for the samples of Dehydration, NaCl, SA, MeJA, and ABA treatment, respectively. While for the concatenated sets of ND (NaCl and dehydration) and SMA (SA, MeJA, and ABA), *UBQ*, *HIS*, and *TUA; UBQ*, *eIF* and *Apt* were the three appropriate candidate reference genes, respectively. The results acquired in this study will improve the accuracy of quantification of target gene expression with RT-qPCR analysis in *I. rubscences* and support the importance of selection of suitable reference genes for future gene expression studies in this species.

## Supplementary Information

Supplementary Figures.

Supplementary Table 1.

Supplementary Table 2.

## Data Availability

The sequences data presented in this study are available under NCBI accession numbers OP006153–OP006163 and ON993894. And all the sequences data are also included as Supplementary Table s1.

## Material availability

The seeds of *I. rubescens* were collected from Jiyuan, Henan province. And we statement that *I. rubescens* is not a protecting plant species in China, so we can harvest its seeds or other materials.
