# Supplementary material for: Validation of suitable reference genes by various algorithms for gene expression analysis in Isodon rubescens under different abiotic stresses
Source: Sci Rep. 2022 Nov 15;12:19599. doi: 10.1038/s41598-022-22397-5 (PMC9666634; doi:10.1038/s41598-022-22397-5)
Supplement: Supplementary file 1 — Supplementary Figures. [file 41598_2022_22397_MOESM1_ESM.pdf]

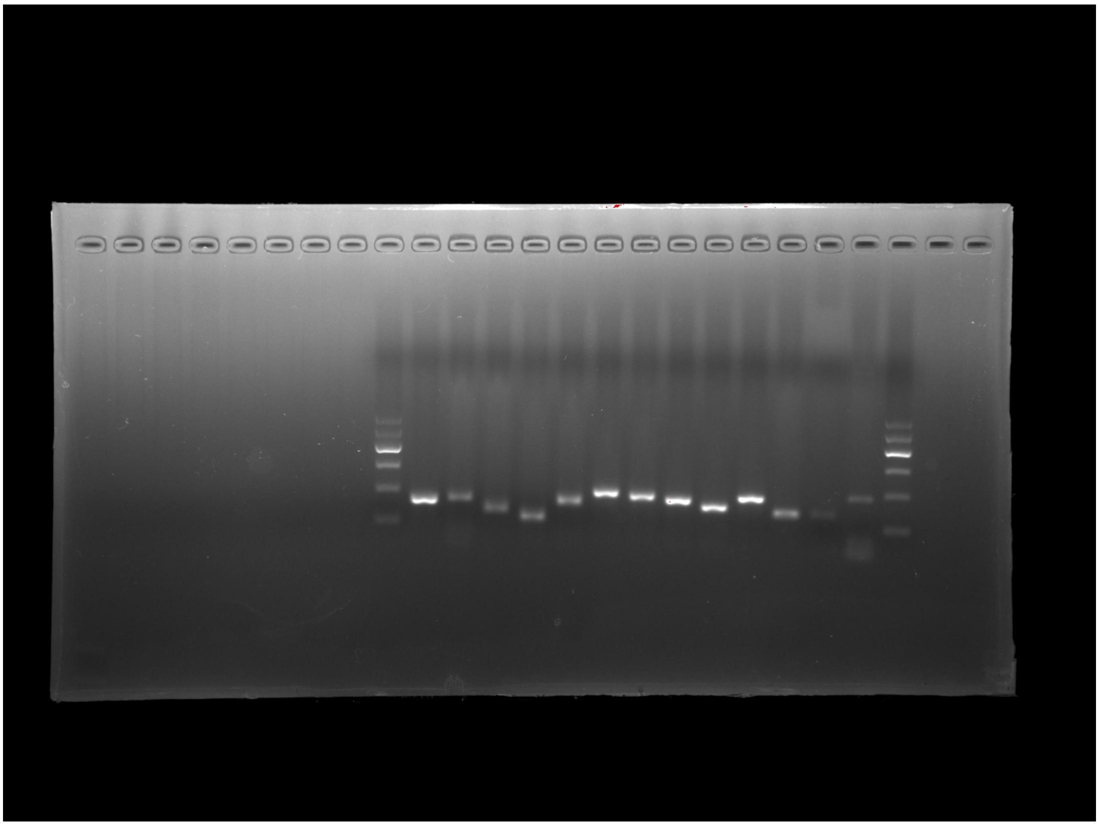

Supplementary Figure S1. The original full-length gel of the Figure 1. The lines from left to right is 600 bp DNA marker, *18S*, *TUB*, *ACT*, *Apt*, *HIS*, *UBQ*, *TUA*, *Cycl*, *GAPDH*, *eIF*, *PP2A*, *EF-1α*. The last one was discarded because of the presence of primer dimers. The first ans the last lanes were 600 bp DNA marker. The penultimate lane was eliminated due to the presence of primer dimers.

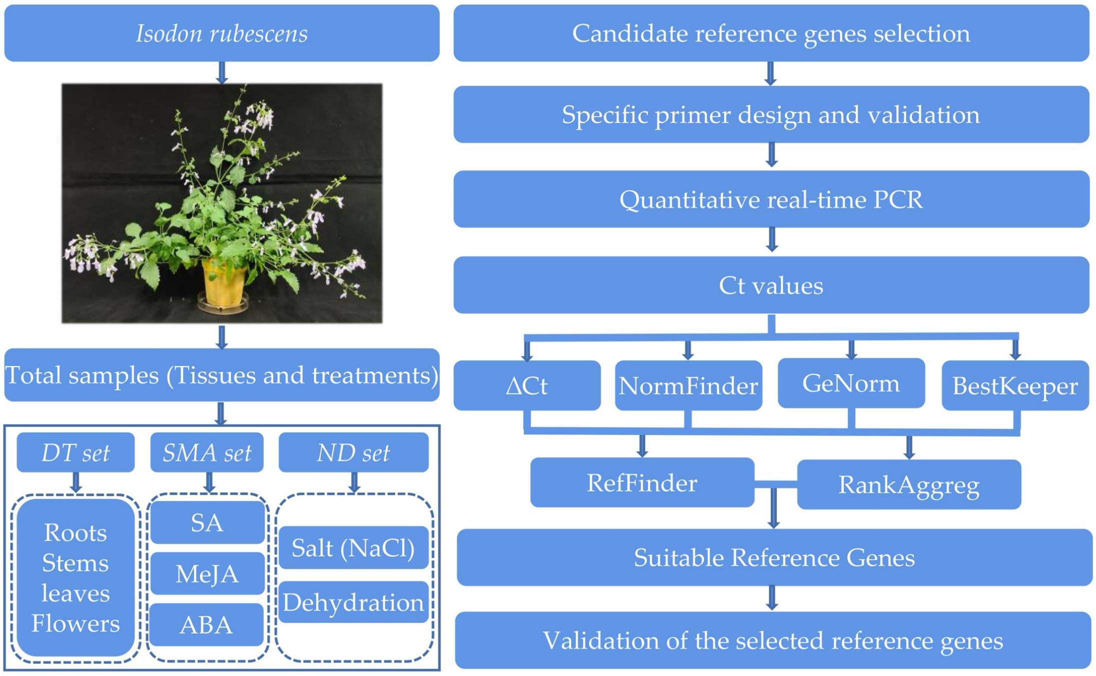

Supplementary Figure S2. Sample sets and data analysis flow chart. DT: The samples of different tissues. ND: The samples of NaCl and dehydration treatments. SMA: The samples of SA, MeJA, and ABA treatments.
